# Selective Growth of GaP Crystals on CMOS-Compatible Si Nanotip Wafers by Gas Source Molecular Beam Epitaxy

**DOI:** 10.1021/acs.cgd.3c01337

**Published:** 2024-03-20

**Authors:** Navid Kafi, Songdan Kang, Christian Golz, Adriana Rodrigues-Weisensee, Luca Persichetti, Diana Ryzhak, Giovanni Capellini, Davide Spirito, Martin Schmidbauer, Albert Kwasniewski, Carsten Netzel, Oliver Skibitzki, Fariba Hatami

**Affiliations:** † Institut für Physik, Humboldt Universität zu Berlin, 12489 Berlin, Germany; ‡ Dipartimento di Fisica, Università di Roma Tor Vergata, 00133 Roma, Italy; § IHP-Leibniz Institut für Innovative Mikroelektronik, Im Technologiepark 25, 15236 Frankfurt (Oder), Germany; ∥ Dipartimento di Scienze, Universita Roma Tre, 00146 Roma, Italy; ⊥ Leibniz Institut für Kristallzüchtung, Max-Born Str.2, 12489 Berlin, Germany; # Ferdinand-Braun-Institut gGmbH, Leibniz-Institut für Höchstfrequenztechnik, Gustav-Kirchhoff-Str. 4, 12489 Berlin, Germany

## Abstract

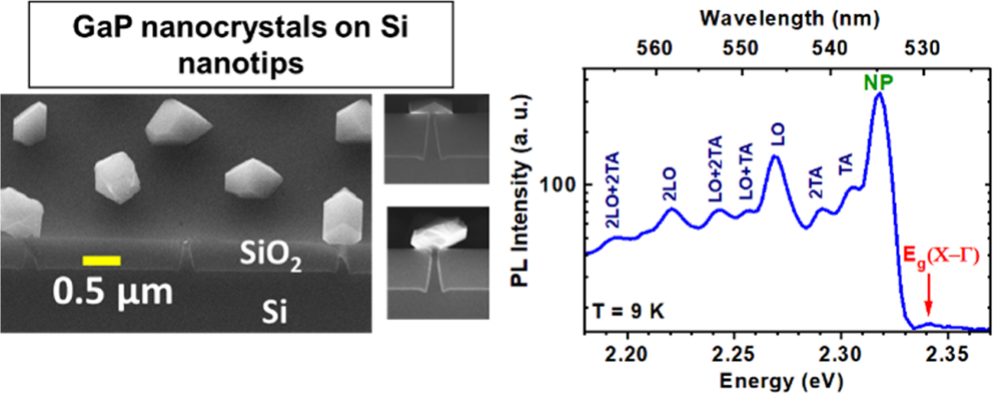

Gallium phosphide (GaP) is a III–V semiconductor with remarkable optoelectronic properties, and it has almost the same lattice constant as silicon (Si). However, to date, the monolithic and large-scale integration of GaP devices with silicon remains challenging. In this study, we present a nanoheteroepitaxy approach using gas-source molecular-beam epitaxy for selective growth of GaP islands on Si nanotips, which were fabricated using complementary metal–oxide semiconductor (CMOS) technology on a 200 mm n-type Si(001) wafer. Our results show that GaP islands with sizes on the order of hundreds of nanometers can be successfully grown on CMOS-compatible wafers. These islands exhibit a zinc-blende phase and possess optoelectronic properties similar to those of a high-quality epitaxial GaP layer. This result marks a notable advancement in the seamless integration of GaP-based devices with high scalability into Si nanotechnology and integrated optoelectronics.

## Abstract 2

Gallium phosphide (GaP) is a III−V semiconductor with remarkable optoelectronic properties, and it has almost the same lattice constant as silicon (Si). However, to date, the monolithic and large-scale integration of GaP devices with silicon remains challenging. In this study, we present a nanoheteroepitaxy approach using gas-source molecular-beam epitaxy for selective growth of GaP islands on Si nanotips, which were fabricated using CMOS technology on 200 mm n-type Si(001) wafers. Our results show that GaP islands with sizes on the order of hundreds of nanometers can be successfully grown on CMOS-compatible wafers. These islands exhibit a zinc-blende phase and possess optoelectronic properties similar to those of high-quality epitaxial GaP layers. This result marks a notable advancement in the seamless integration of GaP-based devices with high scalability into Si nanotechnology and integrated optoelectronics.

## Introduction

Gallium phosphide exhibits the natural zinc-blende (ZB) crystal structure and boasts an indirect room-temperature green band gap of 2.26 eV, a broad transmission range from 0.55 to 11 μm, minimal two-photon absorption for wavelengths beyond 1.1 μm, a relatively high refractive index (*n* = 3.6 at 500 nm), large optical nonlinearity, resulting in strong optical confinement and indicating a large χ^(3)^ nonlinearity, and a noncentrosymmetric crystal structure, leading to a nonzero piezo-electric effect and large χ^(2)^ nonlinearity. Due to its distinctive properties, GaP stands out among other III–V materials, rendering it an optimal choice for both active and passive optoelectronic devices. Its characteristics make it particularly well suited for applications in the visible (VIS) and infrared (IR) spectral ranges, enabling high-power operation across all telecommunications bands. (For a review of GaP photonics, refer to ref (1).) Additionally, GaP possesses advantageous mechanical properties, making it resistant to mechanical strain and harsh weather conditions. Furthermore, GaP distinguishes itself as the III/V semiconductor with the closest lattice constant to silicon (Si), exhibiting a negligible lattice mismatch of less than 0.4%. Consequently, it is the optimal choice as a buffer layer for the seamless monolithic integration of other III–V epilayers and quantum structures into the Si technology.

Although GaP is an indirect semiconductor like Si, isoelectronic doping of GaP with impurities such as N or O can enable light emission.^2^ For this reason, GaP has been the first III–V semiconductor used in the production of low-efficiency light-emitting diodes (LEDs).^3^ The emission performances of GaP-based LEDs can be boosted by embedding quantum structures such as InP and GaAs quantum wells and dots in GaP.^4−6^ The other possibility to develop GaP light emitters is the modification of the crystal structure of GaP from the zinc-blende phase to the wurtzite phase (WZ), for example, by facilitating nanowires, which are characterized by a pseudodirect band gap in the green range.^7−9^ It has also been demonstrated that GaP nanowires have potential applications as emitters and waveguides in nanodevices, such as those involving neuron adhesion and biosensing.^10,11^ Nanostructured GaP membranes can be used as frequency converters, enabling the conversion of visible light to telecom wavelengths,^12,13^ and as optical nanocavities and waveguides within hybrid architectures.^14−17^ Moreover, epitaxial GaP films hold great potential for photovoltaic applications.^18,19^

The combination of all of these possible devices makes GaP a promising material for integrated optoelectronics on Si. However, the monolithic integration of GaP with Si poses challenges, a common issue shared with other III–V semiconductors, owing to the inherent differences in crystal structures, coefficients of thermal expansion, and the formation of polar/nonpolar heterointerfaces between Si and GaP. These factors lead to epilayers featuring a significant density of structural defects, such as stacking faults/microtwins, misfit and threading dislocations, and antiphase domains (APDs),^20,21^ which strongly influence the optoelectronic properties of the devices.

All of these crystal imperfections currently pose obstacles to achieving the desired III–V/Si high-performance, low-cost, and large-scale hybrid devices in microelectronics and integrated optoelectronics.^20−23^

Even though the lattice mismatch between GaP and Si is small, it is still significant, resulting in a low critical epitaxial GaP layer thickness of 64 nm.^21^ Consequently, beyond this critical thickness, threading defects occur in the GaP layer and misfit dislocations form at the interface to Si.

To mitigate misfit and significantly increase the critical layer thickness of the GaP epilayer, various monolithic approaches have been employed. One such method involves reducing the lattice spacing of GaP to match that of Si by introducing nitrogen (N), resulting in a GaP_1–*x*_N_*x*_ alloy that is lattice-matched to Si.^19,22^ Additional strategies include selective growth on nanostructured Si substrate and epitaxy of nanowires.^24^ Moreover, techniques like direct or adhesive wafer bonding^1,25^ and transfer printing of epitaxial layers^13^ have also been employed.

While successful demonstrations validate the efficacy of these approaches, ongoing efforts are necessary to enhance material quality, ensure compatibility with complementary metal–oxide semiconductor (CMOS) technology, and maintain high scalability and low cost. This is crucial for the prospective integration of high-performance GaP-based devices such as light emitters and detectors onto the Si platform.

Limiting the interface area between GaP and Si using nanostructures is a practical approach to suppress the formation of defects. Along this line, nanoheteroepitaxy (NHE)^26^ on nanometer-sized Si tips embedded in SiO_2_ has been proven to be a viable route by enabling the demonstration of selective area growth of different material systems, such as InP/Si using GS-MBE,^27^ GaAs/Si using MOVPE,^28^ and Ge/Si using solid-source MBE.^29,30^ The advantages of NHE include the following:^27−30^ (i) The heteroepitaxial strain energy can be reduced by distributing it in three dimensions through the compliance effect. This can minimize the driving force for plastic relaxation and extended defect formation in the epitaxial layer. (ii) The smaller interface area existing between III–V and Si helps suppress intermixing during growth and annealing, minimizing autodoping. (iii) Owing to the limited lateral extension of the tips, single-step terraces can be observed on the Si seed area, leading to a decrease of the APD density. Furthermore, NHE allows for precise deposition on specific sites, making it ideal for complex photonics and optoelectronics.

In this work, we utilized gas-source molecular-beam epitaxy (GS-MBE) to investigate the selective growth of GaP islands on arrays of Si(001) nanotips (NT) via the nanoheteroepitaxy approach. The tips with a density of (1–6) × 10^8^ per cm^2^ were fabricated on a 200 mm Si wafer under CMOS-compatible conditions. The morphology of the islands was investigated by scanning electron microscopy (SEM) and atomic force microscopy (AFM), while X-ray diffraction (XRD) and Raman spectroscopy were used to examine the structural characteristics. Photoluminescence (PL) spectroscopy was used to investigate the optical properties. Our results demonstrate the selective growth and successful integration of GaP islands with bulklike properties on Si tips wafers with a density higher than 10^8^ islands per cm^2^. This outcome represents a significant step toward integrating GaP-based devices, including light emitters, with high scalability into Si nanotechnology and integrated optoelectronics.

## Methods

The Si NT substrates were fabricated on 200 mm n-type Si(001) wafers in a state-of-the-art pilot line running a 130 nm SiGe BiCMOS technology under CMOS-compatible conditions (for detailed information, see ref (29)). The tips have a top diameter of 20 ± 10 nm and are arranged in square arrays with an area of 1.5 cm^2^ and a tip–tip distance (pitch) of 0.5, 0.8, 1, and 2 μm. Different pitch sizes allow us to explore different sample regions for various experiments. For the examination of an ensemble of the islands, we focused on an array with a small pitch size, while for examining isolated islands, we used the low-density array. Moreover, these patterns facilitate the analysis of island growth dependence on pitch size and hence may offer valuable insights into the dynamics of nanoheteroepitaxy.

In order to fabricate the NT substrate, the on-axis oriented Si(001) wafer was covered with a hard mask consisting of 120 nm thermal SiO_2_ and 20 nm Si_3_N_4_, followed by a 335 nm photoactive resist spin-coated on top of it. The pattern of the NT arrays was defined by lithography using a deep ultraviolet (DUV) scanner. The Si_3_N_4_/SiO_2_ hard mask areas was then etched using reactive ion etching (RIE), subsequently opening the areas without the resist. The remaining resist and Si_3_N_4_ were removed, and an anisotropic RIE dry etching process, which slowly reduces the diameter of the protecting SiO_2_ layer patches, was employed to generate the desired Si NT structures. The exposed Si NTs were then completely covered by SiO_2_. Finally, a chemical–mechanical polishing process was carried out to reduce the SiO_2_ layer thickness and open a circular Si(001) NT surface.

Before the growth process, Si NT substrates underwent a 10 s wet cleaning using a Piranha solution to eliminate organic residues. Subsequently, a 20 s dip in hydrofluoric acid (HF) was performed to remove the native oxide above Si NTs and open the tips. The substrates were promptly loaded in the MBE system to prevent the formation of new oxides. To mitigate residual moisture from wet cleaning, the substrates were heated at 200 °C for 1 h in the loading chamber. Following this, they were transferred into the growth chamber, equipped with a solid Ga source and phosphine (PH_3_) gas source. A subsequent annealing process at 720 °C for 5 min was performed to eliminate any potential remaining native SiO_2_ on the Si NTs.

For growth, the substrate temperature was reduced to the target temperature. Subsequently, the surface was exposed to Ga atoms emanating from an effusion cell, and thermally decomposed PH_3_ products were generated using a cracking cell operated at 920 °C. The Ga beam flux was controlled by adjusting the Ga temperature, and the PH_3_ flux was regulated using mass flow controllers. An ion gauge in the growth chamber measures the background pressure from PH_3_. While we typically use reflection high-energy electron diffraction (RHEED) intensity oscillation to measure the Ga flux and growth rate, the nanotip morphology on the wafer surface makes this technique unfeasible.

Hence, for the growth rate, we rely on the data obtained from the planar epitaxy and will use the corresponding Ga rate instead of the growth rate for further discussion. This is because the growth rate in nanoheteroepitaxy depends not only on the Ga rate but also on other conditions, particularly the substrate temperature.

**Figure 1:**
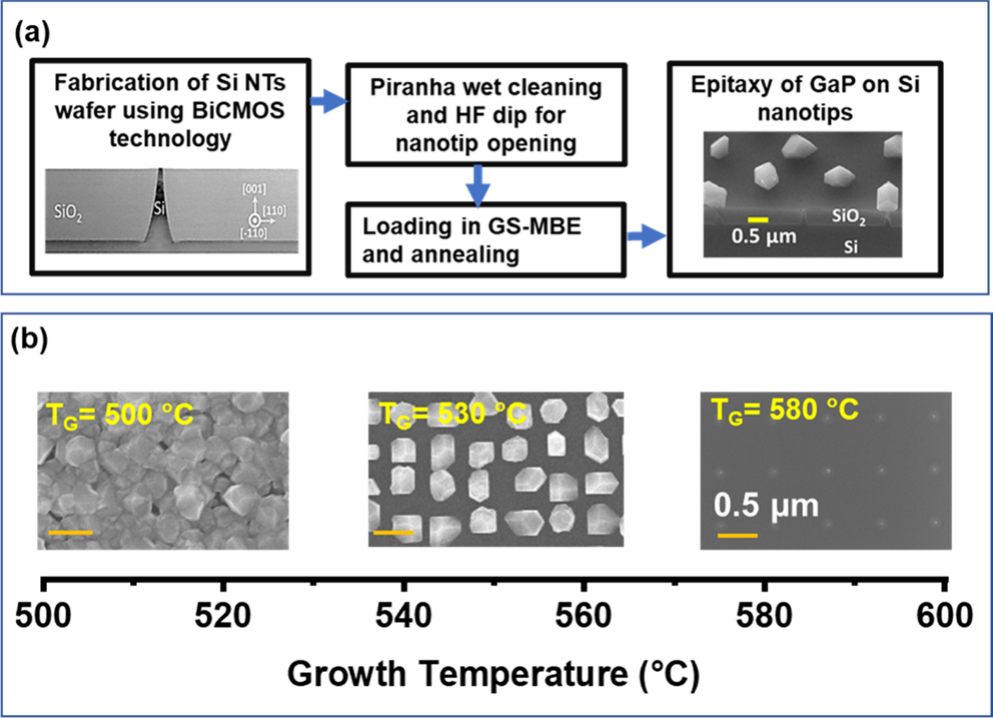
(a) Summary of key processing steps for monolithic integration of GaP on a Si nanotip wafer. The left image presents a cross-sectional transmission electron microscopy (CS-TEM) image of a single tip embedded in SiO_2_, while the right image provides a SEM image with a 45° tilted view of GaP islands grown on Si nanotips. (b) Top-view SEM images of three samples (#1, #2, and #3) grown for 90 min with identical Ga rate and PH_3_ flux but different growth temperatures (*T*_G_). Low growth temperatures result in parasitic growth (left image), while temperatures above 570 °C inhibit growth (right image). Selective growth occurs in the range of temperature between 520 and 570 °C (middle image). All three images have the same scale.

Figure 1(a) illustrates a concise overview of key processing steps involved in the monolithic integration of GaP on Si nanotip wafers using nanoheteroepitaxy.

After growth, the surface morphology was characterized using SEM (SEM Pioneer Two, Raith Nanofabrication) and AFM (Bruker Dimension Icon, Bruker). The AFM measurements were carried out with Bruker Super Sharp TESP-SS cantilevers with a resonance frequency of about 320 kHz and tips featuring a radius of curvature <5 nm in standard tapping mode and under ambient conditions. The high-resolution X-ray diffraction (HR-XRD) measurements were performed on a 9 kW SmartLab system (Rigaku) using Cu Kα_1_ radiation (λ = 1.54056 Å). The GaP crystal phases and their corresponding crystallographic orientations were determined by X-ray pole figures, carried out at selected GaP Bragg reflections using a Theta–Theta X-ray powder diffractometer (GE Sensing & Inspection Technologies) in Bragg–Brentano geometry. Raman measurements were performed at room temperature using a Renishaw inVia system equipped with a diode-pumped solid-state laser at 532 nm, with a grating of 2400 lines/mm, and a Renishaw Centrus 2K2H03 detector with 1040 × 256 pixels. The laser beam was focused on a spot on the sample surface with about 1 μm diameter. The power of the exciting beam was 0.13 mW, and the approximate irradiance was 13 kW/cm^2^. Raman scans were collected with a 50× long-working distance objective. The spectral resolution of the system reached approximately 1 cm^–1^. PL was measured using an argon ion laser (λ = 458 nm) as the excitation source, operating at a power density of 100 mW/cm^2^.

## Results and Discussion

To achieve selective epitaxy of GaP on Si nanotips, we initially determined the optimal growth window, defined by the substrate temperature, PH_3_ flux, and growth rate. This optimized range ensures the appropriate conditions for the selective nucleation of GaP on the Si tips. Selective growth typically requires lower growth rates compared to thin-film growth, promoting adatom diffusion to the tips and their nucleation. The adatoms either absorb on the surface, which is primarily SiO_2_ at the initial stage of growth, and begin to diffuse or undergo the redesorption process. Once adatoms are absorbed, they diffuse to either bind with each other to form new islands or join existing ones.^27,29^ The substrate temperature needs to be sufficiently high to prevent the sticking of GaP on SiO_2_. To determine the optimized growth temperature, we grew a series of samples with a low Ga rate of about 0.5 Å/s, PH_3_ flux of 2.3 sccm, and growth time of 90 min while varying the substrate temperature. Figure 1(b) illustrates the top-view SEM images of three representative samples grown at 500, 530, and 580 °C, all with the same scale. The growth conditions and pitch sizes are summarized in the Supporting Information (Table S1). To enhance clarity in our discussion, these samples are referred to as sample #1, sample #2, and sample #3. The dark gray area in the SEM images indicates the SiO_2_ mask, while the bright gray area shows the GaP or the Si tips. The axis below the SEM images provides the range of the growth temperature. Samples grown at or below 510 °C displayed parasitic growth of GaP on the SiO_2_ mask. This occurrence is attributed to the higher sticking coefficient of Ga on SiO_2_ at lower temperatures, coupled with the low desorption rate of Ga on the “cold” substrate. Consequently, this leads to the accumulation of Ga on the mask surface, promoting parasitic growth. In contrast, at a growth temperature of 580 °C, the desorption of adatoms from SiO_2_ becomes significant, which reduces the growth rate of GaP islands on the Si tips. Hence, the resulting islands are considerably smaller than the islands grown at a lower growth temperature for the same duration. Our results show that the optimal temperature range for selective growth falls between 520 and 570 °C.

**Figure 2:**
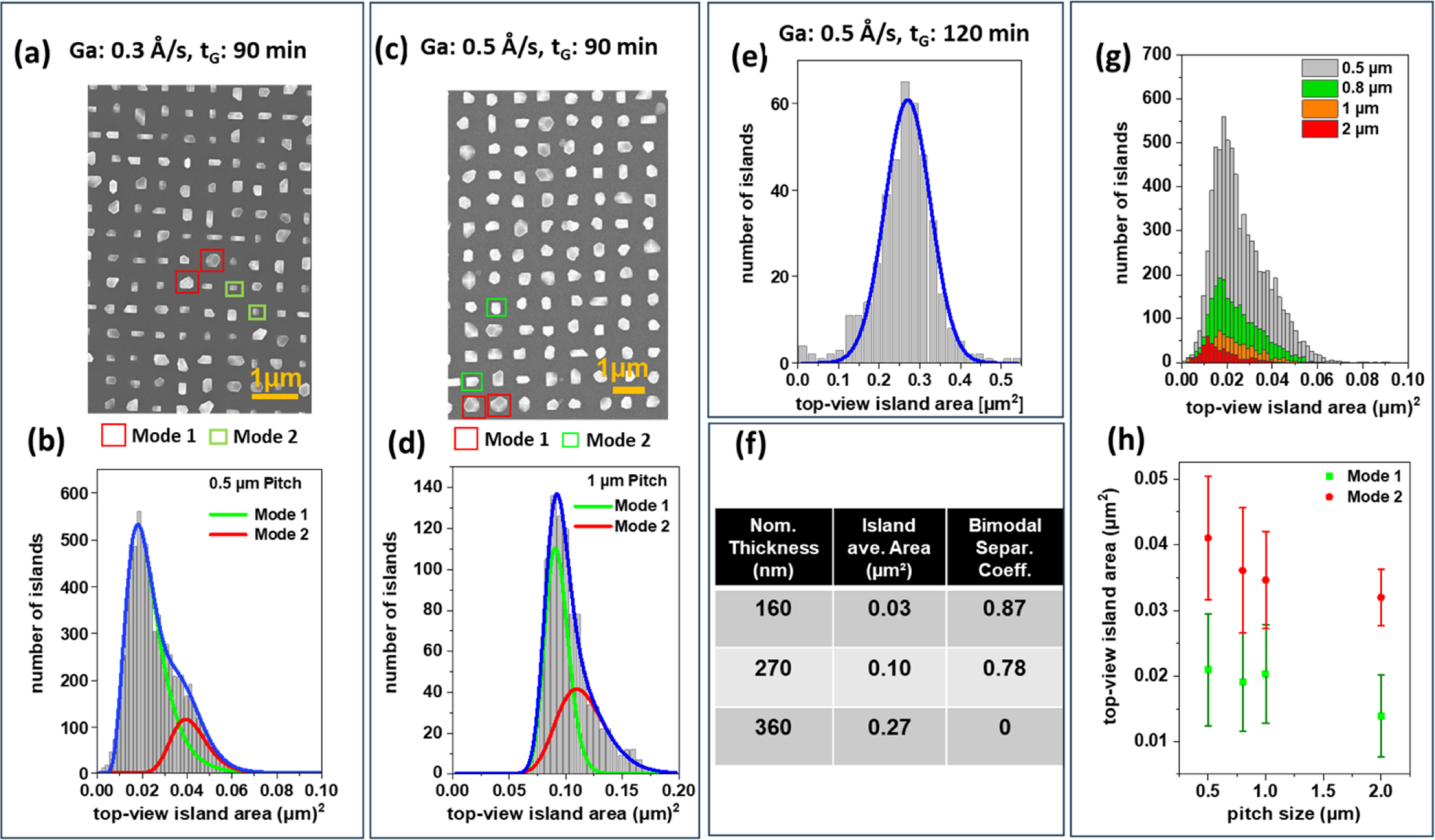
(a) Top-view SEM image of GaP islands on Si nanotips (sample #4) showing various island shapes—some small rectangles (mode 1, green squares) and others larger with multiple facets (mode 2, red squares). (b) Corresponding area histogram and two log-normal fits. (c) Top-view image of sample #2. (d) Histogram of the islands and corresponding fits. (e) Histogram and fit for sample #5. (f) Bimodal separation coefficients for these three samples, along with the corresponding nominal thickness and the averaged island area. (g) Pitch dependence area of GaP islands in sample #4, measured from top-view SEM images and illustrated in histograms. (h) Peak centers and full width at half-maximum (fwhm, bars) obtained by fitting the histograms in (g) using two log-normal fits (modes 1 and 2).

Following the identification of the optimal growth temperature range, we sought a deeper understanding of the dynamics and kinetics involved in nanoheteroepitaxy by investigating samples grown at different rates, times, and pitch sizes. Figure 2 presents the top-view SEM images and size distribution analysis results for three samples grown under varying Ga rates and growth times within the optimized range of substrate temperature. The analysis involved a statistical examination of the size distribution using the top-view SEM images of the samples. A Python script was programmed to determine the area of the islands, which was then organized into histograms. A top-view SEM image of the corresponding sample, which includes hundreds of GaP islands, was provided as input to the program. The program identified the scale bar in the image, determined its length in terms of pixels, and calculated the area per pixel. After that, the top-view area of the islands was identified using the findContours function of the OpenCV library and subsequently put into histograms. For more details, refer to the Supporting Information. The same approach was applied to analyze the influence of tip distance (pitch size) on the growth of the islands. For pitch sizes of 0.5, 0.8, 1, and 2 μm, the corresponding numbers of analyzed islands were 7135, 2320, 770, and 412, respectively. Note that the area of the patterned squares remained constant; therefore, with smaller pitch sizes, there were more islands, resulting in enhanced statistical reliability.

Figure 2(a) shows the top-view SEM images of the sample (sample no. 4) grown at 565 °C using a Ga rate of about 0.3 ± 0.1 Å/s for 90 min, which results in a nominal thickness of about 160 ± 50 nm. The nominal thickness is derived by multiplying the growth rate and growth time. Note that, for thin film growth, we typically use a growth rate in the range of 1–3 Å/s, with the same uncertainty range of ±0.1 Å/s. For a low growth rate, this uncertainly impacts significantly the resulting nominal thickness.

The SEM image suggests the presence of bimodal growth in this sample. Some islands appear to have a rectangular shape and smaller size (mode 1, marked in a green square), while others exhibit more facets and larger size (mode 2, marked in a red square). Figure 2(b) shows the distribution of the top-view area of the islands and the corresponding fit. The best fit for the histogram was achieved using two log-normal distributions,^28^ with corresponding fitted curves peaking at 0.018 and 0.039 μm^2^.

Figure 2(c) shows a representative SEM image of an array of another sample (sample #2) with a pitch size of 1 μm grown at 565 °C with a Ga rate of about 0.5 ± 0.1 Å/s for 90 min (nominal thickness of 270 ± 50 nm). Bimodal growth is again clearly present, but it is less pronounced compared to sample #4 in Figure 2(a). Representatives for modes 1 and 2 are highlighted in red and green, respectively. Figure 2(d) shows the distribution of the top-view area of the islands, with two modes peaked very close at values of 0.09 and 0.10 μm^2^.

In Figure 2(e), the histogram represents the data collected from the SEM image of a sample (sample #5) grown at 545 °C with a Ga rate of about 0.5 ± 0.1 Å/s for 2 h (nominal thickness of about 360 ± 70 nm). The top-view SEM image of the sample is provided in the Supporting Information. The data can be fitted very well using a single Gaussian with a peak at 0.27 μm^2^. Evidently, bimodal growth does not occur for this sample.

To assess the degree of bimodality, we employ the ratio of the integral over the range where the distributions of mode 1 and mode 2 overlap to the integral of the entire distribution (depicted by the blue curves). This ratio provides the overlap ratio, while its complement represents the bimodal separation coefficient. The bimodal separation coefficient indicates the distinctiveness of the two modes in the bimodal distribution relative to the entire distribution. The table in Figure 2(f) summarizes the bimodal separation coefficient for samples #2, #4, and #5, along with the corresponding nominal thickness and the averaged island top area (*A*_ave_). Notably, a larger averaged area for the islands correlates with a smaller bimodal separation coefficient. A correlation between the bimodal growth of large GaAs islands on Si nanotips and the occurrence of twinning within those islands has been reported.^28^ Another explanation for the enhancement of bimodal growth with an increase in the size of islands could be linked to the minimization of the total energy of the crystal. In this scenario, atoms on smaller islands may migrate and coalesce into larger islands (ripening). These larger islands, in turn, possess a lower surface energy and, consequently, exhibit greater thermodynamic stability. An indicator of the reduced surface energy of larger islands is their higher number of facets. However, at this stage, additional experiments are necessary to comprehend the connection between facet numbers and sizes with the occurrence of bimodal growth in GaP islands.

To gain a better understanding of the growth dynamics and the GaP growth rate in nanoheteroepitaxy, we compared the mean height of the islands with the expected nominal thickness. The mean height was measured by using side-view SEM images. The mean height of islands in samples #4 (*A*_ave_= 0.03 μm^2^), #2 (*A*_ave_= 0.10 μm^2^), and #5 (*A*_ave_= 0.27 μm^2^) are approximately 190, 250, and 390 nm, respectively. These values are close to their expected nominal thicknesses. This fact indicates that only adatoms that land in the vicinity of the islands contribute to their growth. This vicinity is defined by the diffusion length of the adatom. Adatoms within only the range of the diffusion length can reach the island and facilitate its growth. This also suggests that most adatoms are desorbed at the beginning of the growth; however, as the size of the islands increases, more adtoms have the chance to be incorporated into islands.^27,29^

It is important to note that the presence of various facets poses challenges in accurately estimating the height from the SEM images. Furthermore, unlike the SEM top-view analysis, height analysis involves only a limited number of islands. Consequently, the estimated height values are less accurate than the top-area values.

We further studied the dependence of the growth outcome on the pitch size between the islands. Figure 2(g) shows the histograms corresponding to data collected from different pitch sizes of sample #4 (SEM image shown in Figure 2(a)). Each histogram was again fitted with two log-normal distributions (mode 1 and mode 2). Figure 2(h) shows the peak centers of the distribution used for the fits. The bars, representing the tolerance range, are estimated based on the full width at half-maximum (fwhm) of the peaks. Despite the overlap between the bars, we see a decrease in the top-view island area with increasing pitch size. The smallest overlap occurs for 2 μm pitch size. This can be explained by the diffusion and exchange of adatoms between islands. Adatoms that land on islands can diffuse along the surface of islands onto the surrounding SiO_2_ mask. There, they can diffuse further and either be recaptured by another island or desorb. A larger pitch size requires a longer travel time, increasing the probability that adatoms will desorb from the surface before reaching other islands. These adatoms are lost to growth; therefore, the islands grown on a silicon nanotip array with a large pitch size are smaller than those grown on an array with a smaller pitch size.

**Figure 3:**
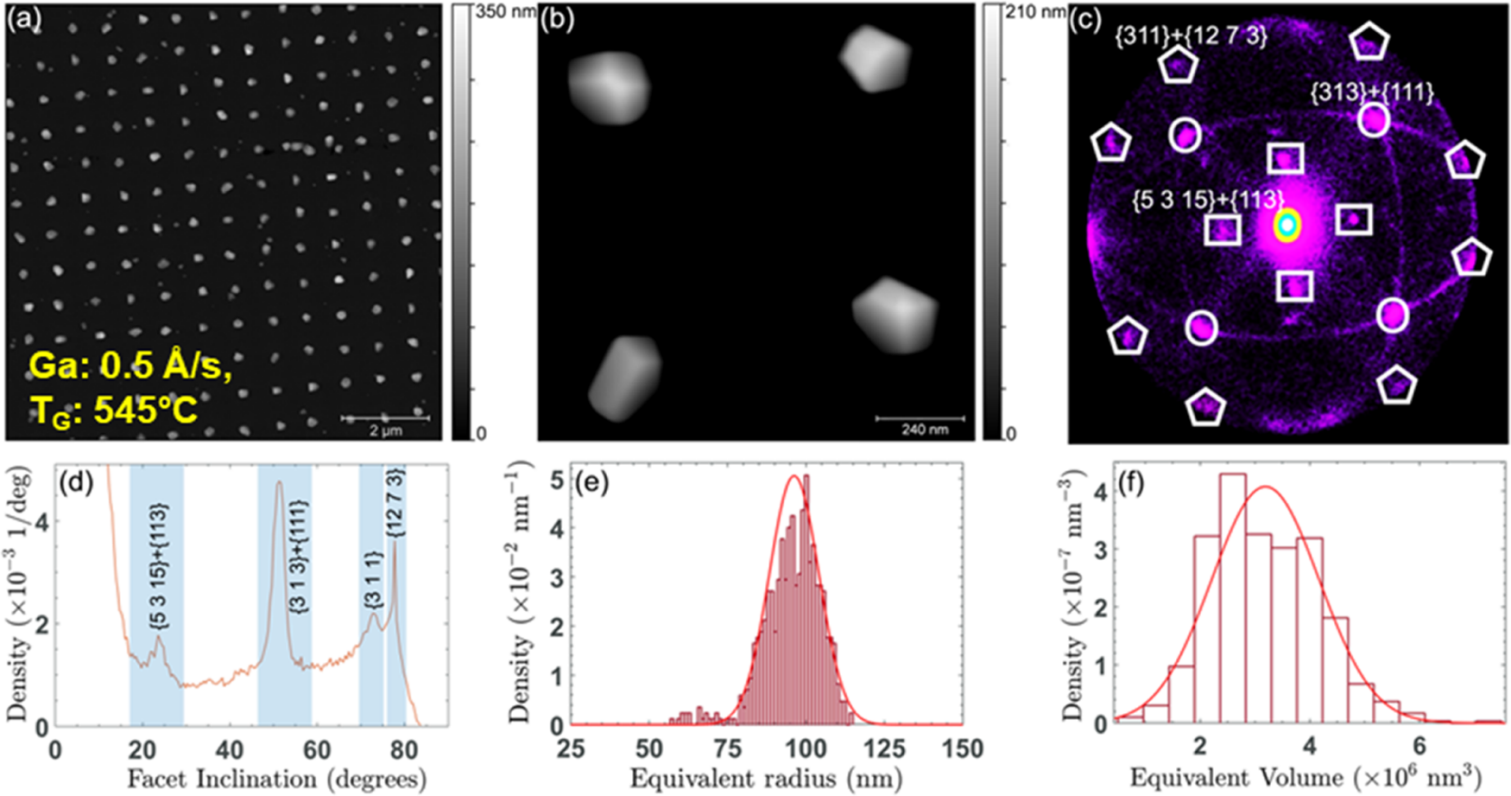
(a) 10 × 10 μm^2^ AFM image of GaP islands (sample #6). (b) Enlarged view showing the individual morphologies of four islands. (c) Facet analysis plot of 600 islands. (d) Cumulative distribution of facet inclinations of these 600 islands. Distribution of (e) equivalent radius and (f) volume of 600 GaP islands.

Using AFM investigation, we quantitatively analyzed the GaP island morphology to assess the facet orientation and their statistical distribution. The 10 × 10 μm^2^ AFM image in Figure 3(a) shows more than 100 GaP islands of a sample grown using a Ga rate of 0.5 ± 0.1 Å/s for 90 min at 545 °C (sample #6). For the analysis, we examined an area with a pitch size of 0.8 μm. This not only ensures a sufficient distance between the islands but also provides an adequate number of islands for more robust statistical analysis. Upon closer inspection of the region depicted in Figure 3(a), a higher-magnification AFM image (Figure 3(b)) unveils the intricate 3D faceting of four individual islands. In order to obtain quantitative information on faceting, we have applied the known facet plot (FP) analysis.^31^ FP consists of a two-dimensional (2D) diagram, where the position of each spot represents the local normal orientation relative to the substrate plane, while the intensity shows the relative amount of the surface with that orientation. The center of the diagram refers to the substrate orientation. This analysis encompassed multiple 10 × 10 μm^2^ regions, totaling more than 600 islands. In Figure 3(c), the FP diagram reveals the presence of three distinct facet families: the shallowest, marked by rectangles, corresponds to angles of 21–25° with respect to the (001) direction and can be identified with the {5 3 15} and {113} facets. Another set of FP loci, forming angles of 47–54° from (001), is highlighted by circles and is associated with {313} and {111} facets. At last, ultrasteep facets are observed at angles of 72–78° from (001), i.e., {311} and {12 7 3} orientations, and are evidenced by pentagons in the FP. Following this, the statistical distributions of facet inclination, radius, and volume (see Figure 3(d)–(f)) were examined for these 600 islands. Figure 3(d) presents the cumulative facet inclination distribution (considering the weight of the number of pixels of each facet). We clearly observe peaks corresponding to each set of crystallographic faces discussed in the FP. As typically observed in multifaceted heteroepitaxial islands,^32,33^ the shallower facets are located at the island top, while the steeper facets are at the island base. The islands exhibiting {313} and {111} facets demonstrate the highest density, aligning well with the common understanding that the (111) crystal plane typically possesses the lowest surface energy in zinc-blende GaP, the same as observed in other III–V semiconductors as well.

The size of GaP islands was further studied via statistical analysis of AFM images. Figure 3(e),(f) shows the distribution of the equivalent radius and volume for the 600 islands. The equivalent radius is calculated as the radius of a disc with the same projected lateral area as the area covered by an island. As shown in Figure 3(e), the equivalent radius distribution of GaP islands for this sample peaked at around 96 nm (area of 0.03 μm^2^) with a width of 8 nm. The volume distribution appeared broader and peaked at 3.2 × 10^6^ nm^3^, with a width of 9.8 × 10^5^ nm^3^, as demonstrated in Figure 3(f). To note, despite having the same Ga rate and growth time as sample #2, the islands in this sample were smaller. This difference can be attributed to the higher resolution of the AFM method compared with the SEM and to the significant uncertainty, estimated at around 20%, in the growth rate.

The shape and faceting of the islands may hint at the change in the crystal structure. GaP forms naturally in the zinc-blende phase, but it can be crystallized in the wurtzite phase under stress or inducing crystal defects. In order to investigate the crystal structure of the islands, X-ray diffraction measurements were performed on sample #5. The distribution of its islands is depicted in Figure 2(e).

**Figure 4:**
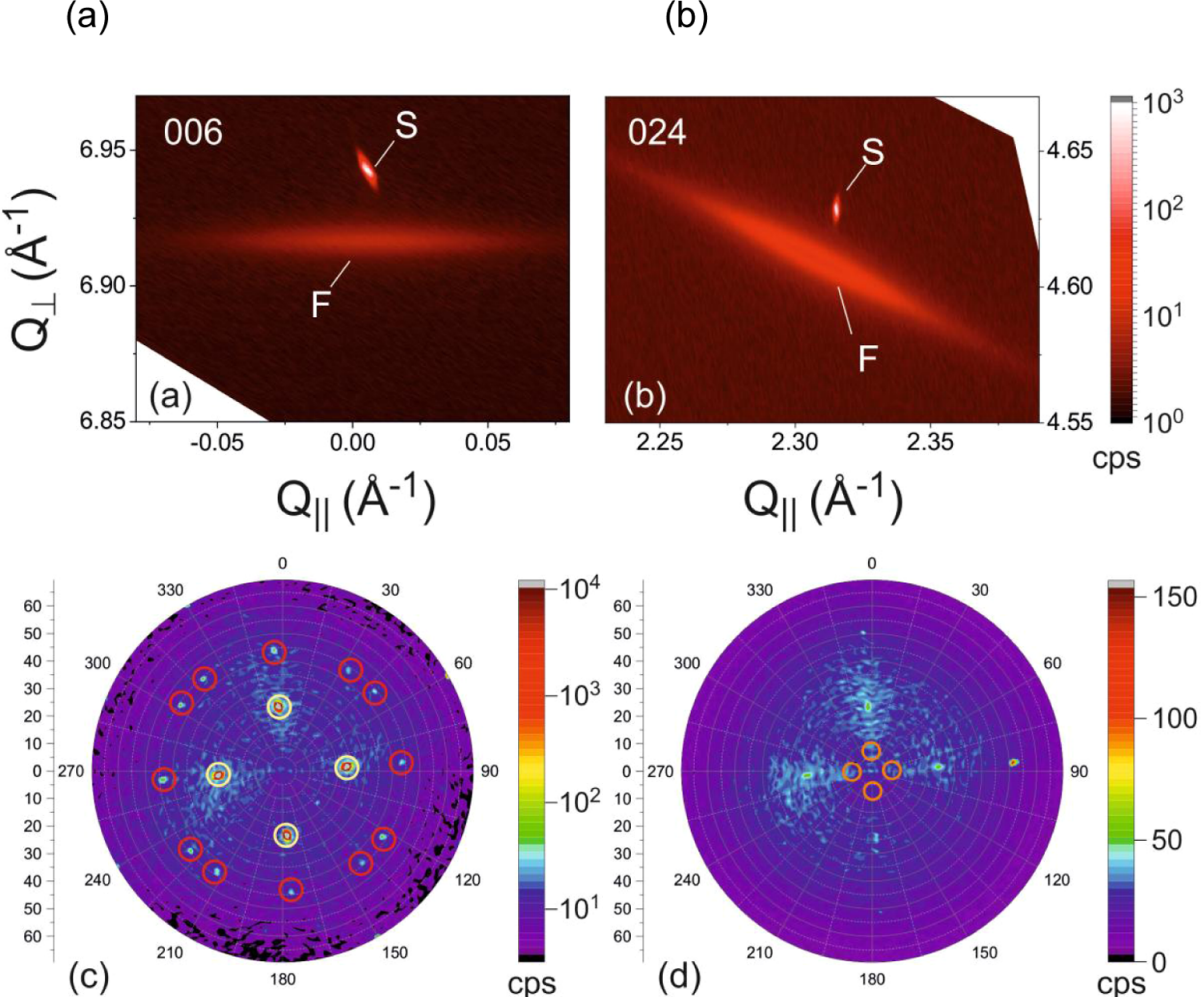
X-ray reciprocal space maps of about 10^6^ islands on sample #5, performed close to the (a) symmetrical 006 and (b) asymmetrical 024 Bragg reflections of Si and ZB GaP, proving the presence of a relaxed zinc-blende phase. X-ray pole figures were recorded at (c) 2θ = 56.042° and (d) 2θ = 30.319°. In (c), yellow circles mark the diffracted signal from GaP/Si, whereas red circles show the diffracted signal from the ZB GaP twins. The orange circles in (d) mark the expected peak positions of the GaP WZ phase.

The X-ray diffraction measurements offer a sturdy statistical basis, achieved through the averaging of a significant number of islands. The number of islands is defined by factors such as the beam size on the sample and the pitch size. Typical values are in the range of approximately 10^6^ islands. Since the lattice spacing and hence the Bragg angles of wurtzite and zinc-blende GaP are close to those of Si, their Bragg peaks might be overlapped, and a simple 2θ–ω scan will not be sufficient to distinguish the crystal phases. Furthermore, the WZ (101) net-planes are tilted from the ZB (001) net-plane. This tilt will lead to strongly suppressed WZ peaks in the 2θ–ω scan. Therefore, to identify multiple phases, the corresponding crystal symmetry, and their mutual orientational relationship, high-resolution X-ray reciprocal space maps (RSMs) were recorded. Figure 4(a),(b) shows two exemplary RSMs recorded in the vicinity of the “basis”-forbidden^34^ but still observable symmetrical 006 and the asymmetrical 024 Bragg reflections of the Si substrate. Close to the sharp Si substrate reflections (marked as “S”), broad and weak intensity features (marked as “F”) are observed, which are caused by the corresponding GaP 006 and 024 zinc-blende Bragg reflections. The average peak positions of features F prove a fully relaxed cubic crystal symmetry with a corresponding lattice parameter of *a* = 5.451 Å, which is in excellent agreement with reported values of bulk GaP (*a* = 5.4505 Å). Complementary XRD pole figures were measured to check the possible existence of twins for zinc-blende GaP. Figure 4(c) shows a pole figure measured at 2θ = 56.042°, which corresponds to a net-plane spacing of 1.639 Å. This measurement is sensitive to the GaP 113 zinc-blende and the Si 113 substrate Bragg reflection. For epitaxial GaP on Si, these two reflections cannot be separated within our resolution limit and appear as single peaks marked by yellow circles in Figure 4(c). However, additional—albeit very weak—peaks can be identified. These are caused by the twinning of ZB GaP through the well-known twinning relationship (001)_ZB_ ⇔ (221̅)_ZB_.^35^ The predicted peak positions of these twins are marked in Figure 4(c) by red circles, depicting that an excellent agreement with the experimentally observed peak positions is obtained. Our experiment thus proves the existence of a small fraction of ZB twins inside the GaP islands. In order to examine whether there is also a contribution of the wurtzite phase inside the GaP islands, we recorded corresponding pole figures at WZ GaP Bragg reflections. An exemplary pole figure measured at 2θ = 30.319° is shown in Figure 4(d). This corresponds to the GaP 10.1 WZ Bragg reflection with a net plane spacing of 2.945 Å. If there is coexistence of ZB and WZ, these phases should share a common (0001)_WZ_/(111)_ZB_ net plane. The (101̅1)_WZ_ net planes should thus include an inclination angle of about 7.5° with respect to the pole (corresponds to (001)_ZB_), as shown in Figure 4(d) (see also ref (36) for similar results obtained on InP nanowires). Owing to the fourfold symmetry of the (001) Si substrate, four of these inclined peaks should be observed. These are marked as orange circles in Figure 4(d); however, at these positions, no peaks can be experimentally observed. Similar pole figures at various 2θ angles were measured (not shown), but no signature of the WZ phase was ever observed.

**Figure 5:**
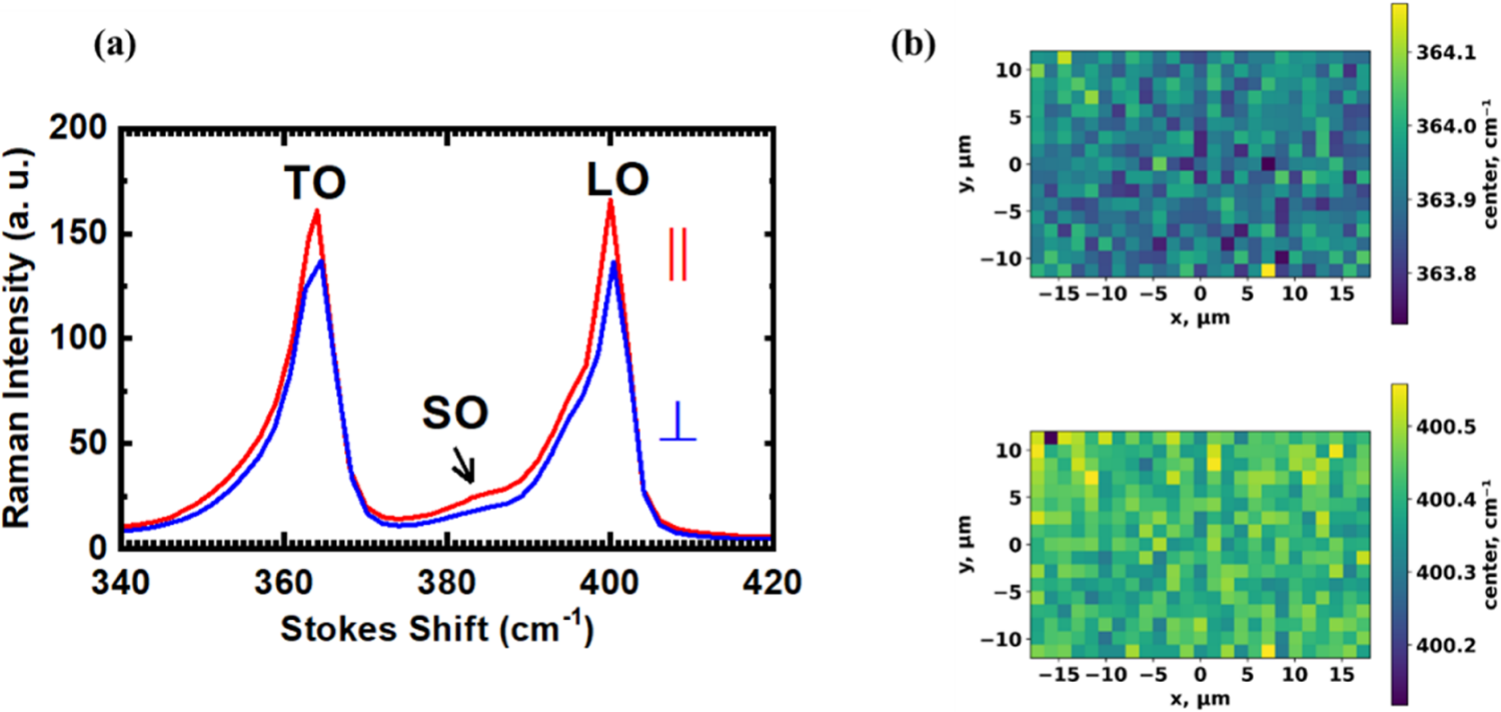
(a) Polarized Raman spectra of GaP islands with parallel and perpendicular polarization configurations (sample #7). (b) Maps of the unpolarized Raman peak position of the GaP islands for the transverse optical (TO, top) and longitudinal optical (LO, bottom) bands.

To investigate the properties of the islands further, we carried out polarized Raman measurements. In Figure 5, the spectrum from a representative sample (sample #7, grown using the same condition as sample #6), measured at 300 K and exited by a 532 nm laser diode, is depicted. For the measurements, an analyzer was mounted at the entrance of the spectrometer, and the polarization of the light was changed in two orthogonal directions, parallel and perpendicular to the analyzer. The sample azimuth was aligned with the [110] direction of the Si substrate parallel to the analyzer. The beam is incident along the [001] direction of the Si. Figure 5(a) displays the Raman spectra for both parallel and perpendicular polarization configurations. We detected two peaks at 363.9 and 400.4 cm^–1^ in both parallel and perpendicular alignments of the polarizer. Note that SiO_2_ is transparent to the green laser light, and therefore, the Raman spectra do not include any information about SiO_2_.

GaP in the WZ and ZB phases exhibits different Raman modes, making Raman spectroscopy a valuable tool for distinguishing between crystal phases. Raman shifts of bulk ZB GaP show two peaks at 367 cm^–1^ (TO) and 403 cm^–1^ (49.9 meV) (LO),^37^ whereas WZ GaP exhibits five peaks at about 78, 356, 363, 395, and 401 cm^–1^.^9,38^ However, due to strain and confinement, the energy of Raman shifts can change. For instance, for ZB GaP nanowires, three Raman shifts at 363, 394, and 401 cm^–1^ have been reported.^9,38^ Comparing our data with the literature, we attribute the Raman shifts measured from GaP islands to the ZB GaP transverse optical (TO) mode at 363.9 cm^–1^ (45 meV) and the longitudinal optical (LO) mode at 400.4 cm^–1^ (49.6 meV). A third weak peak is observed between the TO and LO modes at around 385 cm^–1^, similar to the reported Raman shifts for ZB GaP nanowires. We assign this peak to the surface optical (SO) mode.^39^ The wavenumbers of the Raman shifts differ slightly from the values for bulk GaP, where values of 367 cm^–1^ (TO) and 403 cm^–1^ (LO) are found^37^ as an effect of phonon confinement.^40^

Regarding the polarization dependence, we note that the TO peak would be suppressed in any configuration in a zinc-blend crystal if the light is incident along the [001] direction and the LO peak would be suppressed in the perpendicular configuration.^41^ Instead, the intensity of both peaks is about 90% in the perpendicular configuration compared to that in the parallel configuration. Moreover, in both configurations, the peak position is the same, and the line shape does not change. All of these observations can be explained by the fact that the islands with multiple facets scatter light in different directions. Consequently, the Raman signal is a combination of signals from various orientations.

To verify the uniformity of the sample, we mapped the peak position in an area of 30 × 20 μm^2^ with a spatial resolution such that there is roughly one island per pixel. The top and bottom images in Figure 5(b) show the unpolarized Raman maps for the center values of 363.9 and 400.4 cm^–1^, respectively. Very little dispersion is found, with values of 363.9 ± 0.1 and 400.4 ± 0.1 cm^–1^ for TO and LO modes, respectively. The results from Raman measurements confirm the XRD analysis that the islands are in the zinc-blende phase and are relatively homogeneous in terms of their crystal structure.

**Figure 6:**
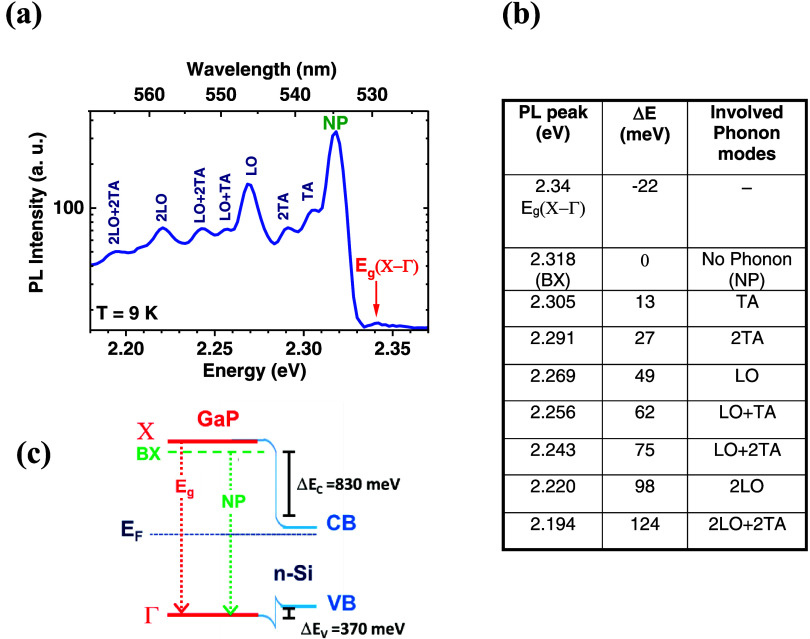
(a) PL spectrum of the as-grown sample with GaP islands at 9 K (sample #5). The sample was exited using a 458 nm laser with a power density of 100 mW/cm^2^. The peak at 2.34 eV is attributed to the indirect band gap, E_g_(X−Γ). The other peaks correspond to the bound-exciton (BX) with no phonon (NP) contribution and phonon replicas.^39^ The longitudinal optical (LO) and transverse acoustic (TA) modes are identified. (b) Table listing the energy positions and origins of the PL peaks, along with the energy difference to NP (Δ*E*) and the corresponding involved phonon modes. (c) Valence band (VB) and conduction band (CB) alignment of the GaP/n-Si heterostructure, along with the corresponding energy offsets (Δ*E*_V_ and Δ*E*_C_), and the position of the Fermi Energy (*E*_F_). Two arrows (E_g_ and NP) represent the two possibilities for the radiative recombination of electrons and holes. The values for CB and VB edges are sourced from ref (46).

To investigate deeper into the optical properties and material quality of the GaP islands, we performed photoluminescence measurements. In Figure 6(a), a representative PL spectrum from an as-grown sample (sample #5) at 9 K is presented. Excitation was achieved using a 458 nm laser at a power density of 100 mW/cm^2^. Due to the indirect band gap of zinc-blend GaP, its interband transitions exhibit excitonic characteristics, enabling the investigation of such transitions only at very low temperatures, typically 20 K or below.^2^ The PL spectrum reveals a distinct low-intensity

line at approximately 2.34 eV and several sharply defined emission lines spanning from 2.194 to 2.318 eV. The PL spectrum is similar to those of high-quality epitaxial GaP bulk materials.^42^ The line at 2.34 eV corresponds to the transition of the indirect X−Γ band gap of zinc-blende GaP, E_g_(X−Γ). As mentioned above, the indirect transitions are usually excitonic in nature, and at low temperatures, momentum conservation involves only the emission of phonons. No-phonon (NP) emission with a sharp line from the recombination of excitons bound to the isolated nitrogen impurities unintentionally introduced during the MBE process and emissions from optical and acoustic phonon replicas are observed.^43^ Even a concentration of N atoms lower than 10^16^ in cm^3^ is enough to show a clear fingerprint of the bound exciton (BX) in the PL spectrum.^2^ The energy of the lines between 2.194 and 2.318 eV fits perfectly to the excitonic transition bound to the isoelectronic nitrogen trap in zinc-blende GaP and phonon replicas. The energy difference between E_g_(X−Γ) and the no-phonon bound exciton at 2.318 eV is about 22 meV, which agrees with the reported binding energy of N-bound excitons in GaP.^44^Figure 6(b) lists the PL energies and the energy difference between the NP emission and phonon replicas. The energy difference Δ*E* is the emitted energy of involved phonons near the X points in the Brillouin zone (TA: 13 meV, LO: 49.9 meV^45^). The energy of the LO mode is in line with the results of Raman spectroscopy.

To get a better picture of the emission lines, the band alignment for the valence (VB) and conduction (CB) bands of the GaP/n-Si heterostructure is illustrated in Figure 6(c). To determine the band alignment and the corresponding conduction band (CB) and valence band (VB) offsets, strain effects were disregarded due to the negligible lattice mismatch between Si and GaP, which is less than 0.4%. The conduction and valence band edge values were sourced from ref (46), providing a conduction band offset of 830 meV and a valence band offset of 370 meV.^46^ GaP was not intentionally doped, leading us to assume that the Fermi level position is in the middle of the band gap of GaP. Conversely, Si was doped with an electron concentration of approximately 10^17^ cm^–3^, shifting the Fermi level closer to the conduction band edge. As the Fermi energies of Si and GaP do not align, charge transfer induces band bending at the heterointerface. Two arrows (E_g_ and NP) display two possibilities for the radiative recombination of electron–hole pairs in zinc-blende GaP. Our PL results confirm the XRD and Raman data that the GaP islands have a zinc-blende crystal structure with properties similar to those of a high-quality epitaxial GaP layer.

## Conclusions

Our work demonstrated the nanoheteroepitaxy approach for the monolithic integration of GaP on CMOS-compatible silicon (001) NTs wafers using gas-source molecular-beam epitaxy. Selective growth of GaP islands with an equivalent radius of approximately 100–200 nm and a height of 100–400 nm on Si NTs was achieved. Various techniques such as scanning electron microscopy, atomic force microscopy, X-ray diffraction, Raman spectroscopy, and photoluminescence were used to characterize the morphology, crystal structure, and optical properties of the resulting GaP islands. The distribution of side facets and the volume of polygonal islands were also investigated by AFM. Pole figure XRD and Raman spectra confirmed the formation of GaP islands in the zinc-blende phase with twinning inside. The PL of the islands was found to be similar to that of the high-quality homoepitaxial GaP layer. The successful selective growth of GaP islands on a 200 mm Si wafer fabricated using CMOS technology offers valuable insights for the seamless monolithic integration of GaP-based devices into nanoscale Si technology for integrated optoelectronics and photonics with large scalability.

## Supporting Information Available

Representative SEM image of sample #5, explanation of the Python script used for the statistical analysis, and a table summarizing all samples discussed in this work (PDF)

## References

[ref1] Wilson D. J.; Schneider K.; Hönl S.; Anderson M.; Baumgartner Y.; Czornomaz L.; Kippenberg T. J.; Seidler P. Integrated gallium phosphide nonlinear photonics. Nat. Photonics 2020, 14, 57–62. 10.1038/s41566-019-0537-9.

[ref2] Thomas D. G.; Hopfield J. J. Isoelectronic traps due to nitrogen in gallium phosphide. Phys. Rev. 1966, 150, 680 10.1103/PhysRev.150.680.

[ref3] Gorton H. C.; Swartz J.; Peet C. Radiative recombination in gallium phosphide point-contact diodes. Nature 1960, 188, 303–304. 10.1038/188303b0.

[ref4] Hatami F.; Masselink W. T.; Harris J. S. Colour-tunable light-emitting diodes based on InP/GaP nanostructures. Nanotechnology 2006, 17, 3703 10.1088/0957-4484/17/15/014.

[ref5] Hatami F.; Lordi V.; Harris J. S.; et al. Red light-emitting diodes based on InP/GaP quantum dots. J. Appl. Phys. 2005, 97, 096106 10.1063/1.1884752.

[ref6] Golz C.; Dadgostar S.; Masselink W. T.; Hatami F. Thermal behavior and carrier injection of GaAs/GaP quantum dots light emitting diodes. Appl. Phys. Lett. 2017, 110, 091101 10.1063/1.4977716.

[ref7] Belabbes A.; Panse C.; Furthmüller J.; Bechstedt F. Electronic bands of III-V semiconductor polytypes and their alignment. Phys. Rev. B 2012, 86, 075208 10.1103/PhysRevB.86.075208.

[ref8] Assali S.; Greil J.; Zardo I.; Belabbes A.; de Moor M. W. A.; Koelling S.; Koenraad P. M.; Bechstedt F.; Bakkers E. P. A. M.; Haverkort J. E. M. Optical study of the band structure of wurtzite GaP nanowire. J. Appl. Phys. 2016, 120, 044304 10.1063/1.4959147.

[ref9] Kang S.; Golz C.; Netzel C.; Mediavilla I.; Serrano J.; Jiménez J.; Hatami F. Gallium phosphide nanowires grown on SiO_2_ by gas-source molecular beam epitaxy. Cryst. Growth Des. 2023, 23, 2568–2575. 10.1021/acs.cgd.2c01447.

[ref10] Hällström W.; Mårtensson T.; Prinz C.; Gustavsson P.; Montelius L.; Samuelson L.; Kanje M. Gallium phosphide nanowires as a substrate for cultured neurons. Nano Lett. 2007, 7, 2960–2965. 10.1021/nl070728e. 17880143

[ref11] Verardo D.; Liljedahl L.; Richter C.; Agnarsson B.; Axelsson U.; Prinz C. N.; Höök F.; Borrebaeck C. A. K.; Linke H. Fluorescence signal enhancement in antibody microarrays using lightguiding nanowires. Nanomaterials 2021, 11, 227 10.3390/nano11010227. 33467141 PMC7829981

[ref12] Rivoire K.; Lin Z.; Hatami F.; Masselink W. T.; Vučković J. Second harmonic generation in gallium phosphide photonic crystal nanocavities with ultralow continuous wave pump power. Opt. Express 2009, 17, 22609–22615. 10.1364/OE.17.022609. 20052186

[ref13] Logan A. D.; Shree S.; Chakravarthi S.; Yama N.; Pederson C.; Hestroffer K.; Hatami F.; Fu K.-M. C. Triply-resonant sum frequency conversion with gallium phosphide ring resonators. Opt. Express 2023, 31, 1516–1531. 10.1364/OE.473211. 36785185

[ref14] Chakravarthi S.; Chao P.; Pederson C.; Molesky S.; Ivanov A.; Hestroffer K.; Hatami F.; Rodriguez A. W.; Fu K.-M. C. Inverse-designed photon extractors for optically addressable defect qubits. Optica 2020, 7, 1805–1811. 10.1364/OPTICA.408611.

[ref15] Gould M.; Schmidgall E. R.; Dadgostar S.; Hatami F.; Fu K.-M C. Efficient extraction of zero-phonon-line photons from single nitrogen-vacancy centers in an integrated gap-on-diamond platform. Phys. Rev. Appl. 2016, 6, 011001 10.1103/PhysRevApplied.6.011001.

[ref16] Englund D.; Shields R. K.; Rivoire K.; Hatami F.; Vučković J.; Park H.; Lukin M. D. Deterministic coupling of a single nitrogen vacancy center to a photonic crystal cavity. Nano Lett. 2010, 10, 3922–3926. 10.1021/nl101662v. 20825160

[ref17] Wu S.; Buckley S.; Schaibley J. R.; Feng L.; Yan J.; Mandrus D. G.; Hatami F.; Yao W.; Vučković J.; Majumdar A.; Xu X. Monolayer semiconductor nanocavity lasers with ultralow thresholds. Nature 2015, 520, 69–72. 10.1038/nature14290. 25778703

[ref18] Feifel M.; Ohlmann J.; Benick J.; Rachow T.; Janz S.; Hermle M.; Dimroth F.; Belz J.; Beyer A.; Volz K.; Lackner D. MOVPE Grown gallium phosphide–silicon heterojunction solar cells. IEEE J. Photovoltaics 2017, 7, 502–507. 10.1109/JPHOTOV.2016.2642645.

[ref19] Dvoretckaia L. N.; Bolshakov A. D.; Mozharov A. M.; Sobolev M. S.; Kirilenko D. A.; Baranov A. I.; Mikhailovskii V. Y.; Neplokh V. V.; Morozov I. A.; Fedorov V. V.; Mukhin I. S. GaNP-based photovoltaic device integrated on Si substrate. Sol. Energy Mater. Sol. Cells 2020, 206, 110282 10.1016/j.solmat.2019.110282.

[ref20] Park J.-S.; Tang M.; Chen S.; Liu H. Heteroepitaxial growth of III-V semiconductors on silicon. Crystals 2020, 10, 1163 10.3390/cryst10121163.

[ref21] Skibitzki O.; Hatami F.; Yamamoto Y.; Zaumseil P.; Trampert A.; Schubert M. A.; Tillack B.; Masselink W. T.; Schroeder T. GaP collector development for SiGe heterojunction bipolar transistor performance increase: A heterostructure growth study. J. Appl. Phys. 2012, 111, 073515 10.1063/1.3701583.

[ref22] Bolshakov A. D.; Fedorov V. V.; Koval O. Y.; Sapunov G. A.; Sobolev M. S.; Pirogov E. V.; Kirilenko D. A.; Mozharov A. M.; Mukhin I. S. Effective suppression of antiphase domains in GaP(N)/GaP heterostructures on Si(001). Cryst. Growth Des. 2019, 19, 4510–4520. 10.1021/acs.cgd.9b00266.

[ref23] Beyer A.; Ohlmann J.; Liebich S.; Heim H.; Witte G.; Stolz W.; Volz K. GaP heteroepitaxy on Si (001): Correlation of Si-surface structure, GaP growth conditions, and Si-III/V interface structure. J. Appl. Phys. 2012, 111, 073515 10.1063/1.4706573.

[ref24] Choi W.; Huang H.-C.; Fan S.; Mohseni P. K.; Lee M. L.; Li X.; Li X. Selective area heteroepitaxy of p-i-n junction GaP nanopillar arrays on Si (111) by MOCVD. IEEE J. Quantum Electron. 2022, 58, 1–6. 10.1109/JQE.2022.3151971.

[ref25] Schneider K.; Welter P.; Baumgartner Y.; Hahn H.; Czornomaz L.; Seidler P. Gallium phosphide-on-silicon dioxide photonic devices. J. Lightwave Technol. 2018, 36, 2994–3002. 10.1109/JLT.2018.2829221.

[ref26] Zubia D.; Hersee S. D. Nanoheteroepitaxy: The Application of nanostructuring and substrate compliance to the heteroepitaxy of mismatched semiconductor materials. J. Appl. Phys. 1999, 85, 6492 10.1063/1.370153.

[ref27] Niu G.; Capellini G.; Hatami F.; Bartolomeo A. D.; Niermann T.; Hussein E. H.; Schubert M. A.; Krause H.-M.; Zaumseil P.; Skibitzki O.; Lupina G.; Masselink W. T.; Lehmann M.; Xie Y.-H.; Schroeder T. Selective epitaxy of InP on Si and rectification in graphene/InP/Si hybrid structure. ACS Appl. Mater. Interfaces 2016, 8, 26948–26955. 10.1021/acsami.6b09592. 27642767

[ref28] Prieto I.; Kozak R.; Skibitzki O.; Rossel M. D.; Zaumseil P.; Capellini G.; Gini E.; Kunze K.; Dasilva Y. A. R.; Erni R.; Schroeder T.; von Känel H. Bi-modal nanoheteroepitaxy of GaAs on Si by metal organic vapor phase epitaxy. Nanotechnology 2017, 28, 135701 10.1088/1361-6528/aa5ec4. 28240990

[ref29] Niu G.; Capellini G.; Schubert M. A.; Niermann T.; Zaumseil P.; Katzer J.; Krause H. M.; Skibitzki O.; Lehmann M.; Xie Y. H.; von Känel H.; Schroeder T. Dislocation-free Ge nano-crystals via pattern independent selective Ge heteroepitaxy on Si nano-tip wafers. Sci. Rep. 2016, 6, 22709 10.1038/srep22709. 26940260 PMC4778127

[ref30] Lange F.; Ernst O.; Teubner T.; Richter C.; Schmidbauer M.; Skibitzki O.; Schroeder T.; Schmidt P.; Boeck T. In-plane growth of germanium nanowires on nanostructured Si (001)/SiO_2_ substrates. Nano Futures 2020, 4, 035006 10.1088/2399-1984/ab82a0.

[ref31] Rastelli A.; von Känel H. Surface evolution of faceted islands. Surf. Sci. 2002, 515, L493–L498. 10.1016/S0039-6028(02)01998-2.

[ref32] Persichetti L.; Sgarlata A.; Fanfoni M.; Balzarotti A. Heteroepitaxy of Ge on singular and vicinal Si surfaces: Elastic field symmetry and nanostructure growth. J. Phys.: Condens. Matter 2015, 27, 253001 10.1088/0953-8984/27/25/253001. 26021279

[ref33] Gradwohl K.-P.; Benedek P.; Popov M.; Matković A.; Spitaler J.; Yarema M.; Wood V.; Teichert C. Crystal habit analysis of LiFePO_4_ microparticles by AFM and first-principles calculations. CrystEngComm 2022, 24, 6891–6901. 10.1039/D2CE00788F.

[ref34] Zaumseil P. High-resolution characterization of the forbidden Si 200 and Si 222 reflections. J. Appl. Crystallogr. 2015, 48, 528–532. 10.1107/S1600576715004732. 25844081 PMC4379439

[ref35] Neubert M.; Kwasniewski A.; Fornari R. Analysis of twin formation in sphalerite-type compound semiconductors: A model study on bulk InP using statistical methods. J. Cryst. Growth 2008, 310, 5270–5277. 10.1016/j.jcrysgro.2008.09.163.

[ref36] Kamath A.; Skibitzki O.; Spirito D.; Dadgostar S.; Mediavilla Martínez I.; Schmidbauer M.; Richter C.; Kwasniewski A.; Serrano J.; Jimenez J.; Golz C.; Schubert M.; Tomm J. W.; Niu G.; Hatami F. Monolithic integration of InP nanowires with CMOS fabricated silicon nanotips wafer. Phys. Rev. Mater. 2023, 7, 103801 10.1103/PhysRevMaterials.7.103801.

[ref37] Mooradian A.; Wright G. B. First order Raman effect in III–V Compounds. Solid State Commun. 1966, 4, 431–434. 10.1016/0038-1098(66)90321-8.

[ref38] da Silva B. C.; Couto O. D. D. Jr.; Obata H. T.; Lima M. M.; Bonani F. D.; Oliveira C. E.; Sipahi G. M.; Iikawa F.; Cotta M. A. Optical absorption exhibits pseudo-direct band gap of wurtzite gallium phosphide. Sci. Rep. 2020, 10, 7904 10.1038/s41598-020-64809-4. 32404930 PMC7221080

[ref39] Gupta R.; Xiong Q.; Mahan G. D.; Eklund P. C. Surface optical phonons in gallium phosphide nanowires. Nano Lett. 2003, 3, 1745–1750. 10.1021/nl034842i.

[ref40] Richter H.; Wang Z. P.; Ley L. The one phonon Raman spectrum in microcrystalline silicon. Solid State Commun. 1981, 39, 625–629. 10.1016/0038-1098(81)90337-9.

[ref41] Aggarwal R.; Ingale A. A.; Dixit V. K. Elucidating the interfacial nucleation of higher-index defect facets in technologically important GaP/Si (001) by azimuthal angle-resolved polarized Raman spectroscopy. Appl. Surf. Sci. 2021, 554, 149620 10.1016/j.apsusc.2021.149620.

[ref42] Hatami F.; Masselink W. T.; Schrottke L.; Tomm J. W.; Talalaev V.; Kristukat C.; Goñi A. R. InP quantum dots embedded in GaP: Optical properties and carrier dynamics. Phys. Rev. B 2003, 67, 085306 10.1103/PhysRevB.67.085306.

[ref43] Alawadhi H.; Vogelgesang R.; Ramdas A. K.; Chin T. P.; Woodall J. M. Indirect transitions, free and impurity-bound excitons in gallium phosphide: A revisit with modulation and photoluminescence spectroscopy. J. Appl. Phys. 1997, 82, 4331 10.1063/1.366241.

[ref44] Merz J. L.; Faulkner R. A.; Dean P. J. Excitonic molecule bound to the isoelectronic nitrogen trap in GaP. Phys. Rev. 1969, 188, 1228 10.1103/PhysRev.188.1228.

[ref45] Dean P. J. Absorption and luminescence of excitons at neutral donors in gallium phosphide. Phys. Rev. 1967, 157, 655 10.1103/PhysRev.157.655.

[ref46] Van de Walle C. G. Band lineups and deformation potentials in the model-solid theory. Phys. Rev. B 1989, 39, 1871 10.1103/PhysRevB.39.1871. 9948405

