# Supplementary material for: Selective Growth of GaP Crystals on CMOS-Compatible Si Nanotip Wafers by Gas Source Molecular Beam Epitaxy
Source: Cryst Growth Des. 2024 Mar 20;24(7):2724–33. doi: 10.1021/acs.cgd.3c01337 (PMC10995952; doi:10.1021/acs.cgd.3c01337)
Supplement: Supplementary file 1 — cg3c01337_si_001.pdf [file cg3c01337_si_001.pdf]

Supplementary Information

Selective growth of GaP crystals on CMOS compatible Si nanotips wafer by gas source molecular beam epitaxy

Navid Kafi[1], Songdan Kang[1], Christian Golz[1], Adriana Rodrigues-Weisensee[1], Luca Persichetti[2], Diana Ryzhak[3], Giovanni Capellini[3,4], Davide Spirito[3], Martin Schmidbauer[5], Albert Kwasniewski[5], Carsten Netzel[6], Oliver Skibitzki[3], Fariba Hatami[1*]

[1]: Institut für Physik, Humboldt Universität zu Berlin, 12489 Berlin, Germany
[2]: Dipartimento di Fisica, Università di Roma Tor Vergata, 00133 Roma, Italy
[3]: IHP-Leibniz Institut für Innovative Mikroelektronik, Im Technologiepark 25, 15236 Frankfurt (Oder), Germany
[4]: Dipartimento di Scienze, Universita Roma Tre, 00146 Roma, Italy
[5]: Leibniz Institut für Kristallzüchtung, Max-Born Str.2, 12489 Berlin, Germany
[6]: Ferdinand-Braun-Institut gGmbH, Leibniz-Institut für Höchstfrequenztechnik, Gustav-Kirchhoff-Str. 4, 12489 Berlin, Germany

E-mail: *hatami@physik.hu-berlin.de

Supporting information for the paper:

Fig SI-1: Representative SEM image sample for sample #5

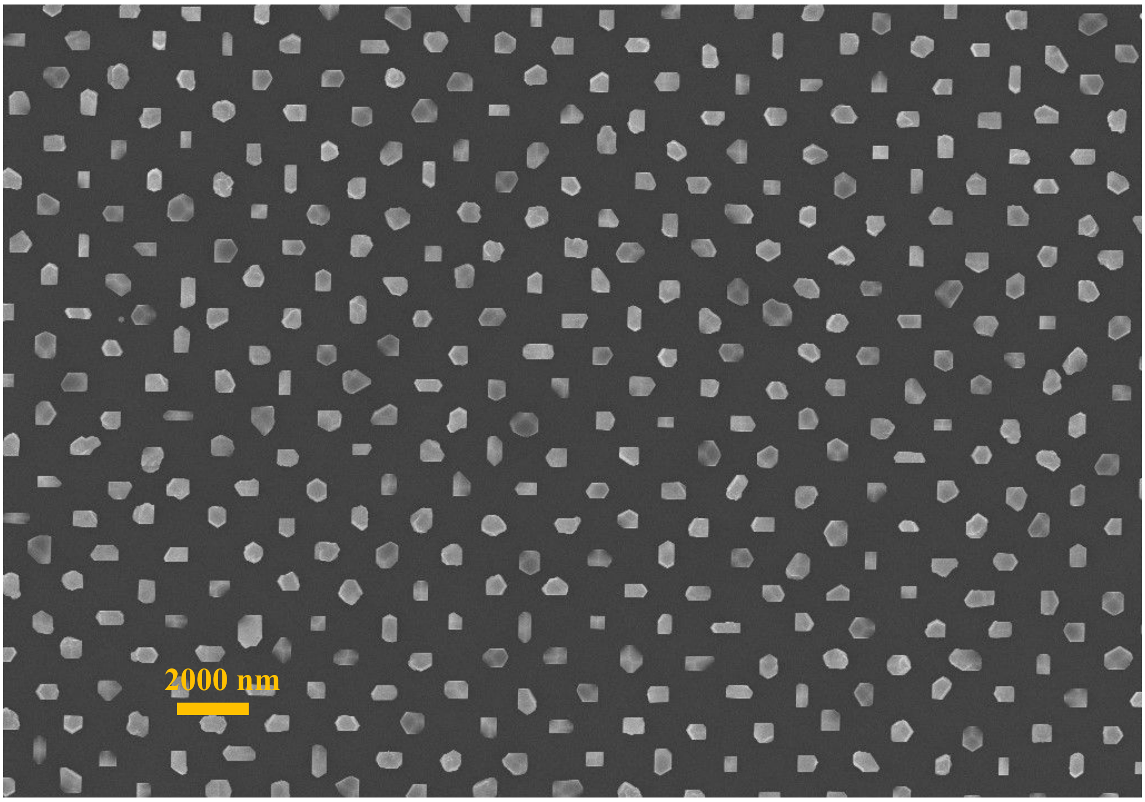

Explanation of the python script used in the statistical analysis of nanocrystals.

The python script operates as follows.

1.) A top-view SEM image of several nanocrystals is given as input. The program finds the scalebar on the image and determines its length in terms of pixels. Together with the scale bar value (e.g. 500 nm) the area per pixel is calculated.
2.) The SEM image is converted into a grayscale image. The user then adjusts the contrast such that the nanocrystals appear white while the background appears black.
3.) Taking the adjusted grayscale image as input, the findContours function of the open source computer vision library detects the contours of all nanocrystals in the image. The function also saves the number of pixels within each contour. The top-view area of an individual island is calculated by multiplying the number of pixels with the area per pixel from step 1.) The area of each island is saved inside a NumPy array.
4.) From this array, the histogram can be constructed and the number of bins can be set.

Compared to manually measuring the top-view area of the nanocrystals, this script is significantly faster and more accurate. Inaccuracies may occur in step 2 since this step requires the adjustment to be done by the user. However, this is expected to be a small error. Another source of inaccuracy is the resolution of the input image, which was generally 1024x720. If the nanocrystal appears to small in the image, its contour will be made up of only a handful of pixels, therefore decreasing the accuracy. To circumvent this, we made sure to take SEM images of a small number of island, to ensure the resolution per nanocrystal was sufficient. To increase the sample size, we analysed several SEM images at different locations on the sample. This script enabled us to quickly measure many crystals with the highest accuracy possible (down to the pixel).

Table 1:

Sample	Characterization method	Figure	Ga rate (Å/s)	PH_3 (sccm)	Growth Temp. (°C)	Growth Time (min)	Pitch (nm)
#1	SEM	1(b)	0.5	2.3	500	90	500
#2	SEM	1(b), 2(c), 2(d)	0.5	2.3	530	90	In 1(b): 500 In 2(a): 1000
#3	SEM	1(b)	0.5	2.3	580	90	800
#4	SEM	2(a), 2(b), 2(g), 2(h)	0.3	2.3	565	90	500, 800, 1000, 2000
#5	SEM, XRD, PL	2(e), 4, 6	0.5	2.3	545	120	1400
#6	SEM, AFM	3	0.5	2.3	545	90	1000
#7	SEM, Raman	5	0.5	2.3	545	90	800

Table 1: The table offers a comprehensive overview of the growth conditions and pitch size for the presented samples in this manuscript, including the figure numbers where the samples are featured.
